# AZGP1 activation by lenvatinib suppresses intrahepatic cholangiocarcinoma epithelial-mesenchymal transition through the TGF-β1/Smad3 pathway

**DOI:** 10.1038/s41419-023-06092-5

**Published:** 2023-09-05

**Authors:** Liming Deng, Wenming Bao, Baofu Zhang, Sina Zhang, Ziyan Chen, Xuewen Zhu, Bangjie He, Lijun Wu, Xiaohu Chen, Tuo Deng, Bo Chen, Zhengping Yu, Yi Wang, Gang Chen

**Affiliations:** 1 Department of Hepatobiliary Surgery, The First Affiliated Hospital of Wenzhou Medical University, Wenzhou, 325035 China; 2 Key Laboratory of Diagnosis and Treatment of Severe Hepato-Pancreatic Diseases of Zhejiang Province, The First Affiliated Hospital of Wenzhou Medical University, Wenzhou, 325035 China; 3 The Second Affiliated Hospital, Department of General Surgery, Hengyang Medical School, University of South China, Hengyang, Hunan 421001 China; 4 Department of Pathology, The First Affiliated Hospital of Wenzhou Medical University, Wenzhou, 325035 China; 5 Department of Epidemiology and Biostatistics, School of Public Health and Management, Wenzhou Medical University, Wenzhou, 325035 China; 6 Hepatobiliary Pancreatic Tumor Bioengineering Cross International Joint Laboratory of Zhejiang Province, The First Affiliated Hospital of Wenzhou Medical University, Wenzhou, 325035 China

**Keywords:** Oncogenes, Oncogenes

## Abstract

Intrahepatic cholangiocarcinoma (ICC) is a primary liver malignancy and is characterized by highly aggressive and malignant biological behavior. Currently, effective treatment strategies are limited. The effect of lenvatinib on ICC is unknown. In this study, we found that AZGP1 was the key target of lenvatinib in ICC, and its low expression in ICC cancer tissues was associated with a poor prognosis in patients. Lenvatinib is a novel AZGP1 agonist candidate for ICC that inhibits ICC-EMT by regulating the TGF-β1/Smad3 signaling pathway in an AZGP1-dependent manner. Furthermore, we found that lenvatinib could increase AZGP1 expression by increasing the acetylation level of H3K27Ac in the promoter region of the AZGP1 gene, thereby inhibiting EMT in ICC cells. In conclusion, lenvatinib activates AZGP1 by increasing the acetylation level of H3K27Ac on the AZGP1 promoter region and regulates the TGF-β1/Smad3 signaling pathway in an AZGP1-dependent manner to inhibit ICC-EMT. This study offers new insight into the mechanism of lenvatinib in the treatment of ICC and provides a theoretical basis for new treatment methods.

## Introduction

Intrahepatic cholangiocarcinoma (ICC) is one of the most aggressive and metastatic tumors, accounting for 10–15% of malignant liver tumors, and it seriously threatens human health. SEER database data demonstrated that the incidence of ICC in the United States increased from 1973 to 2012 [1]. Rizvi et al. speculated that the incidence of ICC could increase by as much as 10-fold globally over the next 20 to 30 years [2]. In the early stage of ICC, patients have no obvious clinical symptoms, so most patients are already in the advanced stage at diagnosis, and only 1/3 patients have the chance to undergo surgery [3]. Radical surgery is the only effective treatment for ICC [4], but the 5-year survival rate is only 20–40% [5–7]. However, due to the high invasiveness and metastasis potential of ICC, the 5-year recurrence rate after radical surgery is 50–70% [8]. Gemcitabine combined with platinum is a first-line chemotherapy regimen for ICC, but the response rate is not high [9]. Second-line treatment options are still lacking for most patients [10]. Therefore, exploring new therapeutic targets and novel drugs is imperative for the treatment of ICC.

Lenvatinib is a tyrosine kinase inhibitor that inhibits several signaling pathways, such as PDGFR1-3 and FGFR1-4, and others inhibit tumor cell proliferation [11–13]. REFLECT demonstrated that lenvatinib was noninferior to sorafenib in advanced hepatocellular carcinoma [14]. The drug was approved by both the FDA and the EMA for the treatment of unresectable hepatocellular carcinoma. Currently, only a few studies have reported the role of lenvatinib in ICC. For example, a study reported that the combination of a PD-1 inhibitor and lenvatinib was effective in the treatment of ICC bone metastasis [15]. An ICC patient-derived xenograft (PDX) model was also used to reveal the effect of lenvatinib [16]. However, its role and mechanism in ICC remain unclear.

AZGP1 is a 41 kDa soluble secretory glycoprotein that is mainly expressed in epithelial cells of the breast, liver and gastrointestinal organs and is involved in fertilization, immune regulation and lipid mobilization [17]. Studies have shown that AZGP1 may serve as a prognostic marker for several cancer types [18–23]. It has been reported that AZGP1 inhibits tumor epithelial-mesenchymal transition (EMT) [24–26], inhibits growth and activates apoptosis [27], but the role of AZGP1 in ICC remains unknown.

The high invasiveness and metastasis of ICC are important factors affecting the prognosis of ICC. EMT is a key step for tumor cells to obtain high invasiveness and migration. Some studies have revealed that EMT of tumor cells occurred before the cells enter the circulatory system [28–30]. Therefore, inhibition of tumor invasion and metastasis requires inhibition of EMT. Transforming growth factor-β1 (TGF-β1) is a cytokine that regulates differentiation, proliferation, apoptosis and cell adhesion [31]. Studies have revealed that the TGF-β/Smad pathway is the strongest EMT-induced pathway [32–34], and AZGP1 plays an important role in regulating the TGF-β signaling pathway. B Kong et al. revealed that AZGP1 could regulate the TGF-β/Ras/ERK signaling axis and inhibit EMT in pancreatic cancer [24]. Hua Tian et al. found that AZGP1 regulated the PTEN/Akt and CD44s pathways to inhibit the migration and invasion of liver cancer [26]. MingYi Xu et al. reported that AZGP1 deletion may induce TGFβ1-ERK2 pathway-induced EMT [25]. However, the molecular mechanism of AZGP1 and the TGF-β1/Smad pathway in ICC remains unclear.

In this study, we predicted the potential key target of lenvatinib in ICC by network pharmacology and bioinformatics methods. Next, we explored the expression of this key target in ICC tissues and further studied its clinical significance. Finally, we explored the mechanism by which lenvatinib regulates its key target and subsequently regulates ICC-EMT.

## Material and methods

### Screening of potential key targets of lenvatinib in ICC

RNA-sequencing (RNA-Seq) data and corresponding clinical data of four ICC datasets, including TCGA-ICC (Tumor=30, Normal=9), GSE107943 [35] (Tumor=30, Normal=27), GSE45001 [36, 37] (Tumor=10, Normal=10), and GSE32958 [38] (Tumor=16, Normal=7), were obtained from the TCGA (https://portal.gdc.cancer.gov/) and GEO databases (http://www.ncbi.nlm.nih.gov/geo). Differentially expressed genes (DEGs) were identified using the above four datasets (tumor vs. normal tissue), and genes with an FDR < 0.05 and |log2 FC | >1 (for GSE107943: FDR < 0.05 and |log2 FC | > 2) identified in the four datasets were defined as DEGs and were selected for further study. In addition, the 2D structure of lenvatinib was derived from the PubChem database (https://pubchem.ncbi.nlm.nih.gov/). The target data were extracted from the PharmMapper database (http://www.lilab-ecust.cn/pharmmapper/) and then transformed into the corresponding protein-coding genes using the UniProt database (http://www.uniprot.org/). From this we obtained 131 genes and divided them into 2 groups according to norm fit score, high fitting score genes (0.5≤scores<1) and low fitting genes (0<scores<0.5). Then, we took the intersection of DEGs and target genes of lenvatinib and obtained the final genes. Subsequently, TCGA-ICC (*n* = 30) and GSE107943 (*n* = 30) with complete prognostic information were used to identify differentially expressed key targets with prognostic value by Kaplan‒Meier (KM) survival analysis. The RCSB Protein Data Bank (https://www.rcsb.org/) provided the 3D structure of the protein. Lenvatinib and target protein structures were converted to pdbqt files using AutoDockTools software (v1.5.6). We used AutoDock Vina software (v1.1.2) to perform the analysis and PyMol software (v2.4.0) to visualize and plot the results.

### Patients and clinical samples

Clinical data and tumor samples of patients who underwent hepatectomy and were diagnosed with pathological ICC at the First Affiliated Hospital of Wenzhou Medical University from December 2012 to December 2019 were collected. Seventy-four patients were enrolled after excluding patients with a history of local tumor therapy (such as microwave therapy, radiofrequency therapy, radiofrequency therapy, radiotherapy and chemotherapy) and patients with other coexisting tumors. Fresh tumor tissues and para-cancer tissues from 20 patients with ICC were collected. Demographic and baseline characteristics, including age at diagnosis and sex, were collected. Laboratory results included CEA, AFP, and CA19-9 levels. Clinicopathological features included TNM staging (the eighth edition of the AJCC staging system for intrahepatic cholangiocarcinoma), tumor location, maximum tumor diameter and number, tumor differentiation, lymph node metastasis, vascular invasion and nerve invasion. Laboratory test results were collected within 1 week before surgery. Routine monitoring of all patients was performed, most recently on April 16, 2021. The overall survival (OS) of a patient was calculated from the day of surgery until death or the day of the last follow-up. Recurrence-free survival (RFS) was defined as the time from surgery to first recurrence of ICC or the date of death from any cause.

The Medical Ethics Committee of the First Affiliated Hospital of Wenzhou Medical University approved the use of patient tissue and clinical data for this study, and written informed consent was obtained from each participant.

### Cell culture

Human ICC RBE, OZ, Hccc-9810 and Hucc-T1 cell lines were used in this study. The Hucc-T1 cell line was a gift from Dr. Lewis Roberts (Mayo Clinic, Rochester, MN, USA). The Japanese Collection of Research Bioresources Cell Banks provided OZ, RBE, and Hccc-9810 for this study. Cell lines were validated with short tandem repeats. The cell culture conditions were 37 °C and 5% CO2, and all cells were grown in RPMI-1640 medium (Gibco, USA) containing 10% fetal bovine serum (FBS) and 1% penicillin‒streptomycin solution. Lentiviruses encoding the AZGP1 shRNA (shAZGP1) or AZGP1 overexpression plasmid (OEAZGP1) were used for transduction.

### Migration and invasion assay

Transwell assays were used for cell migration and invasion. Cells were seeded in the upper transwell chamber, and DMEM containing 10% FBS was used as an inducer in the lower chamber. After 24 h of incubation, cells were fixed with 4% paraformaldehyde and stained with crystal violet. For cell invasion assays, Matrigel chambers and cells were purified with 50 mg/l Matrigel (1:9 dilution) inoculated for 24 h with 4% paraformaldehyde, fixed, stained with crystal violet and counted. Each experiment was repeated 3 times.

### Wound scratch assay

Cell migration was assessed by the wound scratch test. ICC cells were seeded into 6-well plates, and confluent cell monolayers were scraped off with a sterile pipette tip. Pictures were taken with an inverted microscope (Nikon, Japan) at the beginning of the experiment and 48 h later. Each experiment was repeated 3 times.

### Real-time quantitative polymerase chain reaction analyses (RT‒qPCR)

Total RNA was extracted with TRIzol (Invitrogen, USA), and RNA samples were transcribed back to cDNA using the PrimeScript RT Master Mix kit (Takara, China). RT‒qPCR was performed using the Real-Time PCR 7500 FAST Quantitative System (Applied Biosystems, Carlsbad, USA). The primers were as follows: AZGP1 (forward: 5’-AGGCAGAACCAGTCTACGTG-3’, and reverse: 5’-GCCGGTCCAGGATATTTTTGC-3’), and GAPDH (forward: 5’-ACCACAGTCCATGCCATCAC-3’, and reverse: 5’-TCCACCACCCTGTTGCTGTA-3’).

### Immunoblotting

We extracted protein from cells and tissues, and we quantified the concentration using the BCA protein detection kit (Beyotime, China). Separated proteins were transferred to polyvinylidene fluoride (PVDF) membranes by adding 10% or 12% SDS‒PAGE. The PVDF membrane was incubated with the primary antibody overnight at 4 °C, followed by 1 h of incubation with horseradish peroxidase-conjugated secondary antibodies. Target proteins were detected using an enhanced chemiluminescence system. Images were acquired using a multifunctional UVP ChemStudio/PLUS imager (Jena, Thuringia, Germany). The antibodies used were as follows: AZGP1, HDAC1, H3K27Ac from Abcam (Abcam, Cambridge, UK); smad3, p-smad3, and GAPDH from CST (CST, Beverly MA, USA); TGF-β1, E-cadherin, N-Cadherin, Vimentin, HDAC2, HDAC3, and HDAC7 from Proteintech (Proteintech, Chicago, USA).

### Immunohistochemistry (IHC)

We used tissue samples from 74 ICC patients for immunohistochemical analysis. The evaluation procedure was as follows: staining range: 0% positive cells, 0; 1–10%, 1 point; 11–50%, 2 points; 51–80%, 3 points; 81–100%, 4 points. The staining intensity score was recorded as follows: 0 for no staining, 1 was light staining, 2 for moderate staining, and 3 for strong staining. Both scores were recorded by two independent two pathologists in double-blind conditions. Staining score = staining area score× staining intensity score. Scores are divided into “-“ (0), “+“ (1–4), “+“ (5 to 8 points) and “+ + +“ (9–12) [23]. According to the final score, 74 patients were divided into a low expression group and a high expression group.

### Immunofluorescence (IF)

ICC cells were inoculated on glass slides for 24 h, and lenvatinib was then added. After an additional 48 h, the ICC cells were permeabilized with 0.5% Triton X-100 for 30 min after fixation with 4% paraformaldehyde. Slides and primary antibodies were then incubated overnight at 4 °C in sealed chamber solution. Cells were incubated at room temperature in the dark for 2 h after the addition of secondary antibody and 4’6-diamino-2-phenylindole (DAPI). Images were obtained by superresolution laser confocal microscopy (A1R + N-SIM + N-Storm, Nikon, Japan).

### Experimental animal models

Animal experiments were performed using BALB/c nude mice (Experimental Animal Center, Wenzhou Medical University, China). Wenzhou Medical University’s Animal Care and Use Committee approved each animal experiment.

The subcutaneous xenograft mouse model was established by sh-AZGP1 OZ cells and OZ cells implanted subcutaneously in the right hind limb of mice. On the 63rd day post xenograft, all mice were sacrificed, and subcutaneous tumor tissue samples were taken for further analysis. Tumor volume = (long diameter × short diameter ^2^)/2.

The fresh excised ICC tumor tissue was cut into 1–2 mm^3^, placed in RPMI medium containing Matrigel, and implanted in the back of nude mice within 1 h. After 2 months, the established PDX in mouse was passaged 3–4 times, and the appearance of a PDX in the third passage was regarded as a successful model establishment. Fourth-generation ICC-PDX mice were used, and the tumor diameter was measured twice a week to monitor tumor growth. The homology of PDX tumor tissue was verified by short tandem repeat analysis and HE staining. When the tumor volume reached 160–220 mm^3^, 10 ICC-PDX mice were randomly selected and divided into two groups: the control group (*n* = 5, saline) and the lenvatinib group (*n* = 5, lenvatinib, 30 mg/kg, gavage, 1 time/d). After 1 month of treatment or when the tumor reached 1500 mm^3^, the mice were sacrificed, and the tumors were removed for further analysis.

### Chromatin immunoprecipitation (ChIP) assay

We used a ChIP detection kit to perform chromatin immunoprecipitation assays on ICC cells that had been treated with lenvatinib. The antibody used for immunoprecipitation was H3K27Ac (Abcam, Cambridge, UK). The following primers were used for the AZGP1 promoter region: 5’-CTGGAGGACAGTCACATTCCC-3’, reverse: 5’-AGGATGCGGCTATTTCTGCTT-3’. Quantification was performed by PCR on a C1000 Thermal Cycler (Bio-Rad). All experiments were performed in triplicate.

### Short tandem repeat (STR)

The DNA was isolated from the original patient tumor and PDX tumors passaged in mice with Axygen’s genome extraction kit and amplified using the 21-STR amplification scheme, and the STR loci and the sex gene Amelogenin were detected on the ABI 3730XL genetic analyzer. The following autosomal STR loci were analyzed for sex determination: D5S818, D13S317, D7S820, D16S539, VWA, TH01, AMEL, TPOX, CSF1PO, D12S391, FGA, D2S1338, D21S11, D18S51, D8S1179, D3S1358, D6S1043, PENTAE, D19S433, PENTAD, D1S1656 and Amelogenin.

### Differential scanning fluorimetry (DSF)

Label-free nanoDSF technology can accurately detect changes in endogenous fluorescence when proteins are thermally and chemically denaturized. Each control capillary consumes 5 µl AZGP1 solution+5 µl buffer, and each experimental sample consumes 5 µl AZGP1 + 5 µl lenvatinib solution. Proteins and small molecules were incubated for 15 min and detected on the computer, and were analyzed using the protein stability analysis system Prometheus NT.48.

### Statistical analyses

Statistical analysis was performed by R program (version 3.6.1) and IBM SPSS 22.0 (SPSS, Chicago, USA). Continuous variables were expressed as the mean ± standard deviation (SD), statistical analysis was used for *t* tests, normally distributed variables were expressed as median (interquartile range, IQR), and statistical analysis was used for the Wilcoxon rank sum test. Categorical variables were expressed as frequencies (%) and compared using the x^2^ test. Several groups were compared using one-way ANOVA. Differences in OS and RFS were analyzed using the log-rank test and the Kaplan‒Meier (K‒M) method. Cox analysis (univariate and multivariate) was used to identify independent factors impacting OS and RFS in patients with ICC as well as to estimate the appropriate hazard ratio (HR) of the 95% confidence interval (CI). *p* < 0.05 was considered to indicate statistical significance for all tests, which were two-sided.

## Results

### AZGP1 is a potential key target of lenvatinib for the inhibition of ICC

**Fig. 1:**
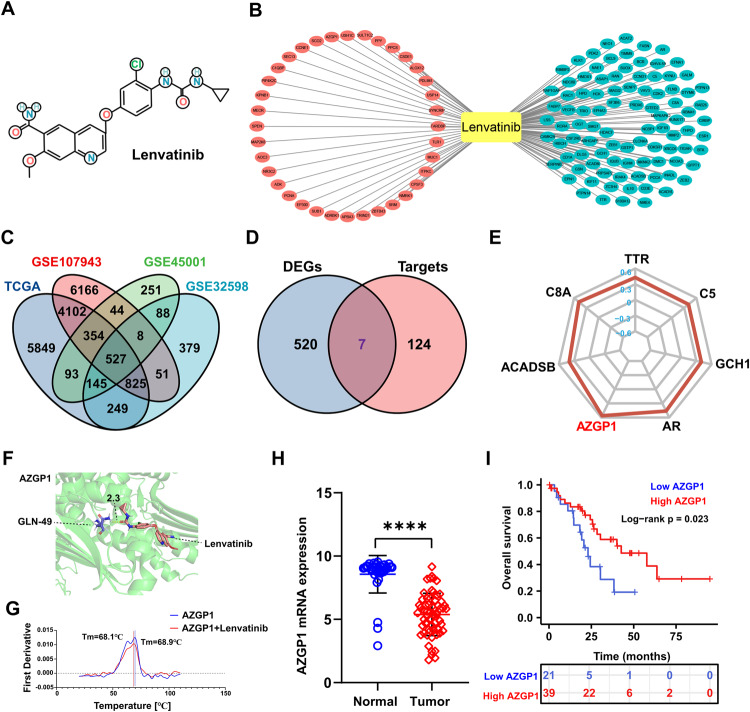
Bioinformatics analysis revealed that AZGP1 is a potential key target of lenvatinib for the inhibition of ICC. **A** Chemical structure of lenvatinib. **B** Lenvatinib key target was obtained by PharmMapper Sever. Red represents high-fitting genes, blue represents low-fitting genes. **C** Common DEGs in TCGA, GSE107943, GSE45001, and GSE32958. **D** Pharmacophore of lenvatinib in ICC. **E** 7-gene Fitscore radar map. **F** Molecular docking pattern diagram of lenvatinib and AZGP1. **G** DSF experiment detected the interaction between lenvatinib and AZGP1. **H** AZGP1 mRNA expression in TCGA-ICC (*n* = 30) and GSE107943 (*n* = 30). **I** Kaplan‒Meier survival curves showed that the OS of ICC patients was superior in the high-expression group compared to the low-expression group in the public database.

The lenvatinib 2D SDF file (Fig. 1A) was downloaded from the PubChem database (PubChem CID: 9823820), and 131 key targets were obtained by PharmMapper Sever (Fig. 1B). In descending order of FIT scores, the top 10 genes were SYNCRIP, USP14, PDLIM1, ALOX12, CSDE1, PPCS, PPY, SULT1C2, USH1C and AZGP1. TCGA, GSE107943, GSE45001, and GSE32958 had 12144, 12077, 1510, and 2272 DEGs (Supplementary Figs. S1A, S1B, S1C, S1D), respectively, and there was a total of 527 DEGs in the four datasets (Fig. 1C). There were 7 candidate targets of lenvatinib in ICC (Fig. 1D): AZGP1, ACADSB, AR, C8A, C5, GCH1 and TTR. According to the fit score, AZGP1 was the gene most related to lenvatinib (Fig. 1E). The possibility of lenvatinib treatment for ICC was verified by molecular docking technique. The docking results showed that the maximum binding free energy of AZGP1 and lenvatinib was −1.65 indicating that that lenvatinib could bind AZGP1 well (Fig. 1F). However, in differential scanning fluorescence (DSF) assay, lenvatinib had no direct interaction with AZGP1 protein. There may be other forms of indirect binding (Fig. 1G). TCGA-ICC (*n* = 30) and GSE107943 (*n* = 30) gene expression profiles and clinical prognostic information were combined into a dataset. AZGP1 expression in cancer tissues was significantly lower than that in paracancerous tissues (Fig. 1H). Based on Kaplan‒Meier survival curve analysis, ICC patients with high AZGP1 expression tended to have better OS than those with low AZGP1 expression (Fig. 1I).

### Decreased AZGP1 expression predicts a poor prognosis in patients with ICC

**Fig. 2:**
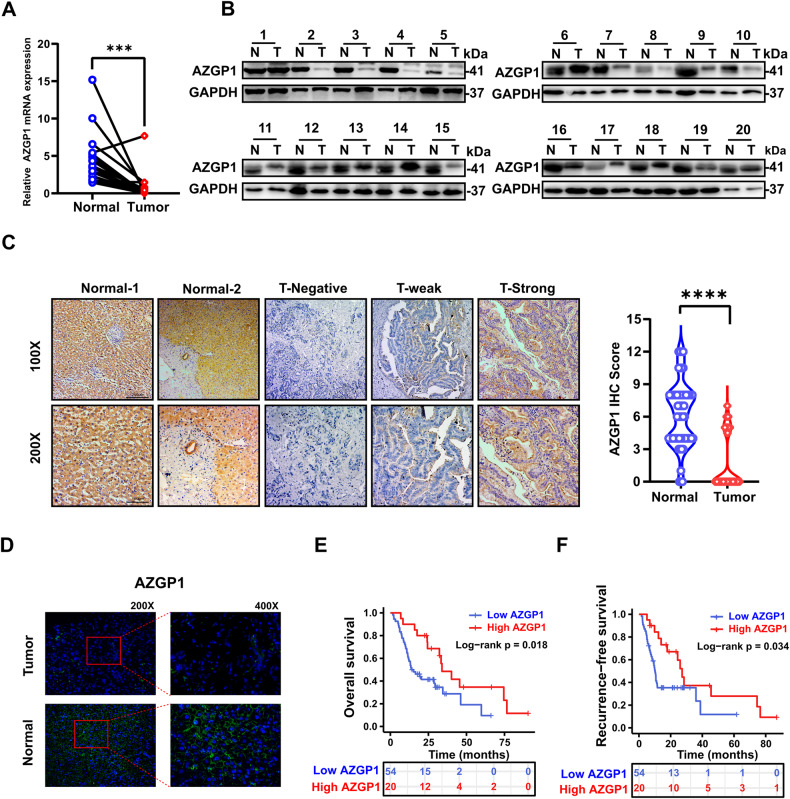
Decreased expression of AZGP1 in ICC patients predicts a poor prognosis. **A** AZGP1 mRNA expression was detected in cancer and adjacent tissues of 20 ICC patients by RT‒qPCR. **B** AZGP1 protein was detected in cancerous and adjacent tissues of 20 ICC patients by Western blotting. **C** The expression of the AZGP1 protein in normal tissue is strongly positive, and is divided into no expression, weak expression, and strong expression in tumor tissue. **D** The expression of AZGP1 was detected in ICC and normal tissues using immunofluorescence. **E** Kaplan‒Meier curves of OS of the AZGP1 expression group based on immunohistochemistry from the in-house cohort. **F** Kaplan‒Meier curves of RFS of the AZGP1 expression group based on immunohistochemistry from the inhouse cohort. ***p < 0.001, and ****p < 0.0001.

RT‒qPCR and Western blotting were applied to assess the expression level of AZGP1 in fresh ICC tissues and indicated that AZGP1 mRNA and protein levels were decreased in ICC tissues vs. normal tissues (Fig. 2A, B). Then, we performed AZGP1 immunohistochemical staining on the tissues of 74 patients with ICC, and semiquantitative scoring under a light microscope was performed by two independent pathologists. AZGP1 protein expression was found to be lower in 72.97% of tumor tissues than in adjacent tissues (Fig. 2C). This result suggested that AZGP1 is downregulated in ICC. The results of tissue immunofluorescence showed that AZGP1 is mainly expressed on the cell membrane (Fig. 2D).

When we analyzed the correlation between the clinical and pathological characteristics of ICC patients and AZGP1 expression, there were no significant with any of the following factors: age, sex, tumor stage, diabetes, tumor size and tumor differentiation (Table 1). Next, we examined the relationship between AZGP1 expression and prognosis in ICC patients. According to Kaplan‒Meier analysis, patients with low AZGP1 expression in ICC had a significantly shorter median OS times than those with high expression (14.5 vs. 33.9 months) (*p* = 0.018, Fig. 2E). In addition, the median RFS in the AZGP1 low-expression group was significantly shorter than that in the AZGP1 high-expression group (10.1 vs. 27.2 months) (*p* = 0.034, Fig. 2F). Among patients with low AZGP1 expression, the 5-year OS and RFS rates were 9.6% and 11.8%, respectively, while the rates for those with high AZGP1 expression were 23.1% and 27.9%, respectively. Our study showed that patients with high AZGP1 expression in ICC lived longer than those with low expression.

**Table 1:** AZGP1 levels and clinicopathological features in 74 ICC patients.

Variables	AZGP1 expression			
	Total (*n* = 74)	Low expression (*n* = 54)	High expression (*n* = 20)	*P* value
Age, years, Mean ± SD	65.81 ± 9.89	65.91 ± 9.81	65.55 ± 10.37	0.891
Gender, *N* (%)				
Male	31 (41.9)	32 (59.3)	11 (55.0)	0.742
Female	43 (58.1)	22 (40.7)	9 (45.0)	
HBsAg, *N* (%)				0.702
Negative	47 (63.5)	35 (64.8)	12 (60.0)	
Positive	27 (36.5)	19 (35.2)	8 (40.0)	
AFP, ng/mL, (IQR)	2.76 (2.1–3.62)	2.93 (2.09–3.7)	2.7 (2.11–3.55)	0.733
CEA, μg/L, (IQR)	2.75 (1.65–7.63)	2.85 (1.65–9.88)	2.25 (1.55–4.18)	0.461
CA19-9, U/mL, (IQR)	104.85 (18.97–711.68)	104.85 (18.97–857.93)	111.65 (9.63–527.9)	0.756
TNM Stage, *N* (%)				
I-II	58 (78.4)	40 (74.1)	18 (90.0)	0.139
III-IV	16 (21.6)	14 (25.9)	2 (10.0)	
Tumor differentiation, *N* (%)				0.588
Well	52 (70.3)	37 (68.5)	15 (75.0)	
Poor	22 (29.7)	17 (31.5)	5 (25.0)	
Tumor location, *N* (%)				
Left	46 (62.2)	29 (53.7)	17 (85.0)	**0.014**
Right	28 (37.8)	25 (46.3)	3 (15.0)	
Tumor size, cm, *N* (%)				0.872
≤5.0 cm	47 (63.5)	34 (63.0)	13 (65.0)	
å 5.0 cm	27 (36.5)	20 (37.0)	7 (35.0)	
Tumor number, *N* (%)				0.48
Single	66 (89.2)	49 (90.7)	17 (85.0)	
Multiple	8 (10.8)	5 (9.3)	3 (15.0)	
Vascular invasion, *N* (%)				0.321
No	57 (77.0)	40 (74.1)	17 (85.0)	
Yes	17 (23.0)	14 (25.9)	3 (15.0)	
Perineural invasion, *N* (%)				0.934
No	56 (75.7)	41 (75.9)	15 (75.0)	
Yes	18 (24.3)	13 (24.1)	5 (25.0)	
Lymph node metastasis, *N* (%)				0.147
No	63 (85.1)	44 (81.5)	19 (95.0)	
Yes	11 (14.9)	10 (19.5)	1 (5.0)	
AZGP1, *N* (%)				
High	20 (27.0)	0 (0.0)	20 (100.0)	
Low	54 (73.0)	54 (100.0)	0 (0.0)	
Vital status, *N* (%)				**0.012**
Alive	35 (47.3)	24 (44.4)	11 (55.0)	
Dead	39 (52.7)	30 (55.6)	9 (45.0)	
Diabetes, *N* (%)				
No	60 (81.1)	41 (75.9)	19 (95.0)	0.063
Yes	14 (18.9)	13 (24.1)	1 (5.0)	

Bold values indicates statistically significant *p* values less than 0.05.

Subsequently, we analyzed the clinicopathological parameters affecting the postoperative survival of patients with ICC. The univariable Cox proportional risk regression analysis showed that tumor differentiation, stage, lymphatic metastasis, CEA level, AZGP1 level and diabetes were the factors influencing the OS of ICC patients after surgery; (*p* < 0.05); tumor differentiation, staging, lymphatic metastasis, CEA level, AZGP1 level, CA19-9 level and diabetes mellitus were the factors influencing postoperative RFS in ICC patients (*p* < 0.05) (Table 2). Multivariate Cox analysis revealed that tumor differentiation and AZGP1 level were independent predictors of OS in patients with ICC (*p* < 0.05); RFS was independently associated with AZGP1 (*p* < 0.05) in patients with ICC (Table 3). Based on these findings, AZGP1 may serve as a useful prognostic factor for ICC patients.

**Table 2:** Prognostic factors in ICC patients by univariate analysis.

Variables	OS		RFS	
	HR (95% CI)	*P*-value	HR (95% CI)	*P*-value
Age>60, year	1.89 (0.80–4.48)	0.145	1.81 (0.76–4.25)	0.179
Gender	1.39 (0.79–2.2.45)	0.25	1.62 (0.91–2.88)	0.101
HBsAg	0.78 (0.42–1.44)	0.424	0.64 (0.35–1.18)	0.158
TNM Stage III, IV	4.65 (2.40–8.99)	**<0.001**	4.03 (2.13–7.63)	**<0.001**
Tumor differentiation	2.23 (1.24–3.99)	**0.007**	1.79 (1.01–3.21)	**0.048**
Tumor size > 5.0, cm	1.42 (0.79–2.56)	0.242	1.35 (0.75–2.42)	0.323
Tumor number	1.53 (0.70–3.31)	0.285	1.55 (0.71–3.36)	0.272
Tumor location	1.16 (0.65–2.08)	0.623	1.09 (0.61–1.97)	0.753
Lymph node metastasis	3.91 (1.84–8.29)	**<0.001**	3.63 (1.74–7.55)	**0.001**
Vascular invasion	1.71 (0.88–3.32)	0.117	1.68 (0.87–3.27)	0.125
Perineural invasion	1.46 (0.78–2.75)	0.236	1.67 (0.89–3.15)	0.111
AFPå 9, ng/mL	1.59 (0.49–5.19)	0.438	1.24 (0.38–4.01)	0.723
CEAå 5, μg/L	2.25 (1.23–4.13)	**0.009**	1.97 (1.09–3.59)	**0.026**
CA19-9å 37, U/mL	1.9 (0.97–3.71)	0.06	2.01 (1.03–3.94)	**0.04**
AZGP1 level	0.44 (0.22–0.89)	**0.021**	0.48 (0.24–0.96)	**0.038**
Diabetes	2.79 (1.38–5.66)	**0.004**	2.04 (1.04–4.02)	**0.039**

Bold values indicates statistically significant *p* values less than 0.05.

**Table 3:** Multivariate analysis using the Cox proportional hazards model.

Variables	HR (95% CI)	*P*-value
OS		
TNM Stage III, IV	3.49 (0.98–12.44)	0.054
Lymph node metastasis	0.87 (0.21–3.59)	0.843
CEA > 5, μg/L	1.42 (0.67–3.01)	0.355
AZGP1	0.45 (0.21–0.96)	**0.038**
Tumor differentiation	2.06 (1.06–4.01)	**0.033**
Diabetes	1.72 (0.76–3.84)	0.182
RFS		
TNM Stage III, IV	2.64 (0.73–9.62)	0.14
Lymph node metastasis	0.73 (0.17–3.21)	0.676
CEA > 5, μg/L	1.18 (0.56–2.47)	0.669
AZGP1	0.46 (0.21–0.96)	**0.04**
Diabetes	1.28 (0.55–2.97)	0.561
CA19-9 > 37, U/mL	1.91 (0.94–3.87)	0.074
Tumor differentiation	1.58 (0.81–3.05)	0.177

Bold values indicates statistically significant *p* values less than 0.05.

### AZGP1 regulates ICC cell proliferation, migration, invasion and EMT

**Fig. 3:**
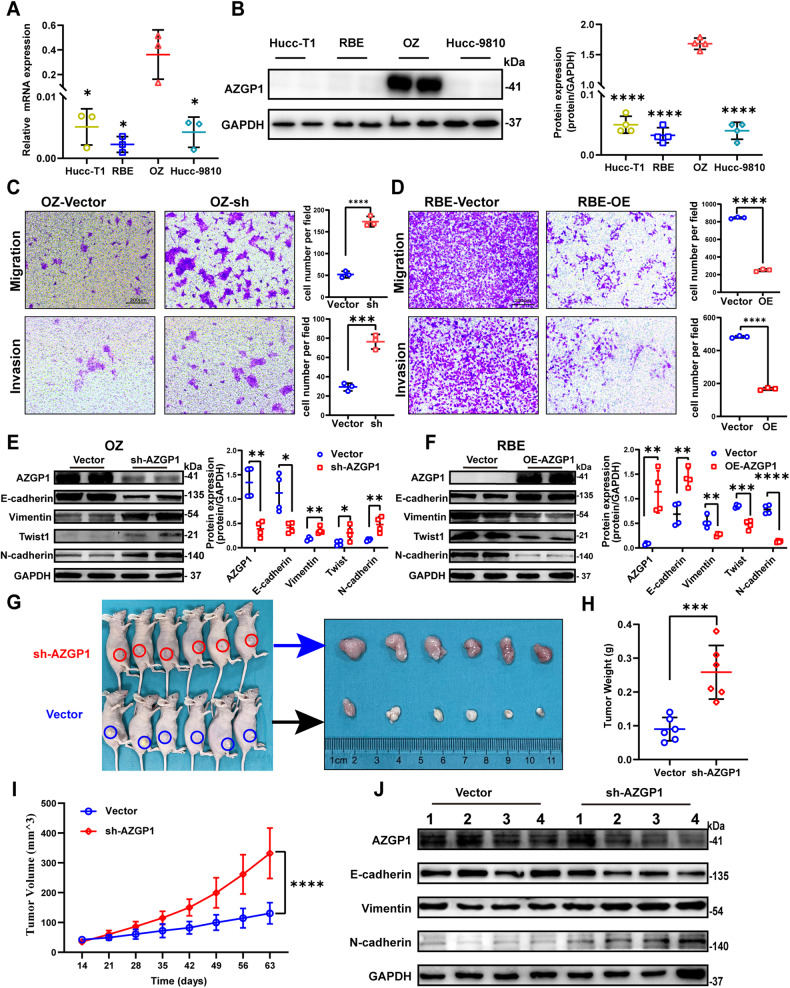
The AZGP1 gene regulates ICC migration, invasion and EMT. **A** AZGP1 mRNA expression levels in Hucc-T1, RBE, OZ and Hccc-9810 cell lines were quantified using RT‒qPCR. **B** AZGP1 protein expression levels in Hucc-T1, RBE, OZ and Hccc-9810 cell lines were detected by Western blotting. **C** AZGP1 knockdown in OZ cells promoted migration and invasion. **D** AZGP1 overexpression in RBE cells inhibited migration and invasion. **E** The protein expression levels of AZGP1, E-cadherin, Vimentin, Twist1, and N-cadherin were detected by Western blotting in OZ cells after AZGP1 knockdown. **F** The protein expression levels of AZGP1, E-cadherin, Vimentin, Twist1, and N-cadherin were detected by Western blotting in RBE cells after overexpression of AZGP1. **G** Xenografts in nude mice showed that OZ cells after AZGP1 knockdown had a higher tumorigenesis effect than the control group. **H** Excised tumor weight. **I** Volume of tumor xenografts in nude mice. **J** Western blotting analysis of E-cadherin, vimentin, and N-cadherin protein expression in the xenograft model. *p < 0.05, **p < 0.01, ***p < 0.001, and ****p < 0.0001.

To confirm whether AZGP1 regulates the biological behavior of ICC cells, the expression of AZGP1 in ICC cell lines was examined. The expression of AZGP1 was the highest in the OZ and lowest in the RBE (Fig. 3A, B). Subsequently, AZGP1 was knocked down in OZ cells and overexpressed in RBE cells, which was confirmed by Western blotting (Supplementary Figs. S2A, S2B). We found that knockdown of AZGP1 significantly promoted colony formation (Supplementary Fig. S2C), migration, and invasion (Fig. 3C, Supplementary Fig. S2E), and EMT (Fig. 3E), while overexpression of AZGP1 significantly inhibited colony formation (Supplementary Fig. S3D), migration, invasion (Fig. 3D, Supplementary Fig. S2F), and EMT (Fig. 3F). Finally, we examined growth and EMT in xenografts after knockdown of AZGP1 in vivo. We found that knockdown of AZGP1 promoted the growth of xenografts in nude mice (Fig. 3G, H, I, Supplementary Fig. S2G). Furthermore, knockdown of AZGP1 promoted the expression of EMT in vivo (Fig. 3J). It is speculated that the expression of AZGP1 is closely related to the tumorigenicity of ICC cells.

### Lenvatinib inhibits ICC proliferation, migration, invasion, and EMT

**Fig. 4:**
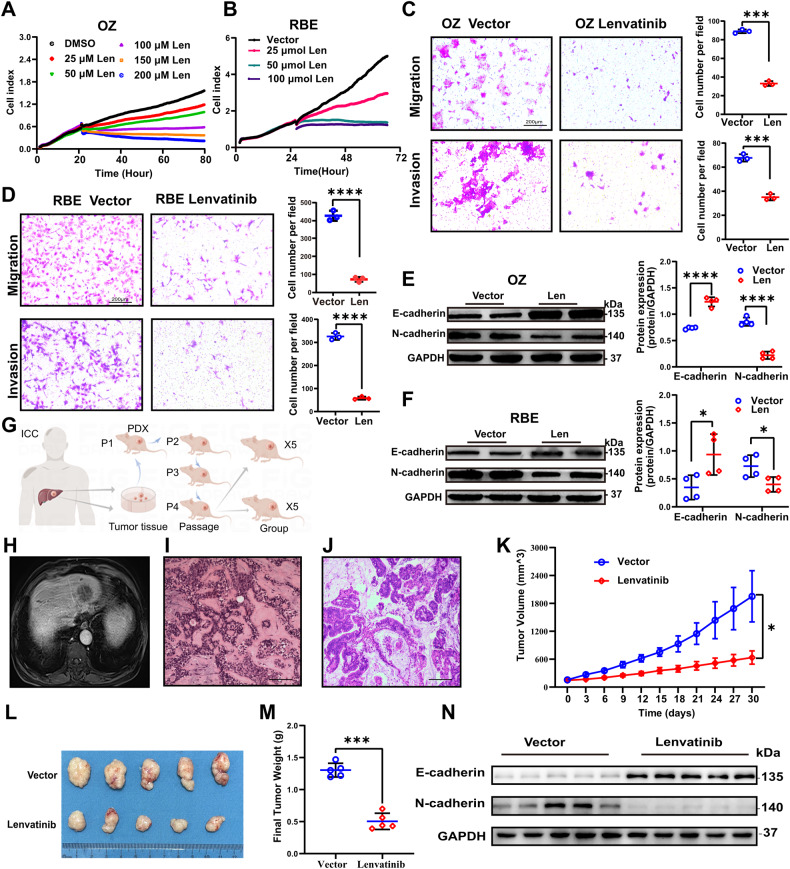
Lenvatinib inhibits ICC cell migration, invasion, and EMT. **A**, **B** RTCA confirmed that lenvatinib inhibited the proliferation of OZ and RBE cells. **C**, **D** Lenvatinib inhibited the migration and invasion of OZ and RBE cells. **E**, **F** The expression levels of E-cadherin and N-cadherin in OZ and RBE cells were detected by Western blotting. **G** Establishment of the model diagram of the ICC-PDX model. **H** CT images of tumors in ICC patients. **I**, **J** Hematoxylin-eosin staining analysis showed that the PDX model tumor were homologous to the patient’s tumor. **K**–**M** Tumor volume and tumor weight changed after lenvatinib treatment. **N** E-cadherin and N-cadherin protein expression in the PDX model was detected by Western blotting. *p < 0.05, ***p < 0.001, and ****p < 0.0001.

RTCA and colony formation assays were used to examine how lenvatinib affects ICC cell proliferation. Lenvatinib had antiproliferative activity on OZ and RBE cells (Fig. 4A, B, Supplementary Fig. S3A, S3B). The lenvatinib IC50 values in RBE and OZ cells were 45.8 μM and 102 μM, respectively. We also examined the effect of lenvatinib on ICC cell migration, invasion and EMT capacity. After exposure to lenvatinib, ICC cells exhibited delayed wound healing (Supplementary Fig. S3C, S3D), and migration and luminal invasion were suppressed (Fig. 4C, D). Western blotting demonstrated that the expression of E-cadherin was increased and the expression of N-cadherin was decreased (Fig. 4E, F). We examined the effect of lenvatinib on ICC carcinogenesis using a PDX model (Fig. 4G). STR analysis showed that the genotype match between the PDX tumor and the original patient tumor was 91% (Supplementary Fig. S3E). Further AFP and CK19 staining indicated that the PDX tumor and the original patient tumor were cholangiocarcinoma, not hepatic cell carcinoma (Supplementary Fig. S3H). H&E staining also showed that the PDX tumor tissue was homologous to the patient’s tumor tissue (Fig. 4H, I, J). We found that lenvatinib inhibited tumor growth in the PDX model (Fig. 4K, L, M, Supplementary Fig. S3F, S3G, S3I). Additionally, lenvatinib inhibited EMT in the PDX model (Fig. 4N). Considering all these results, lenvatinib shows great promise in the field of ICC drug therapy.

### Lenvatinib inhibits ICC EMT by mediating the TGF-β1/Smad3 pathway through AZGP1

**Fig. 5:**
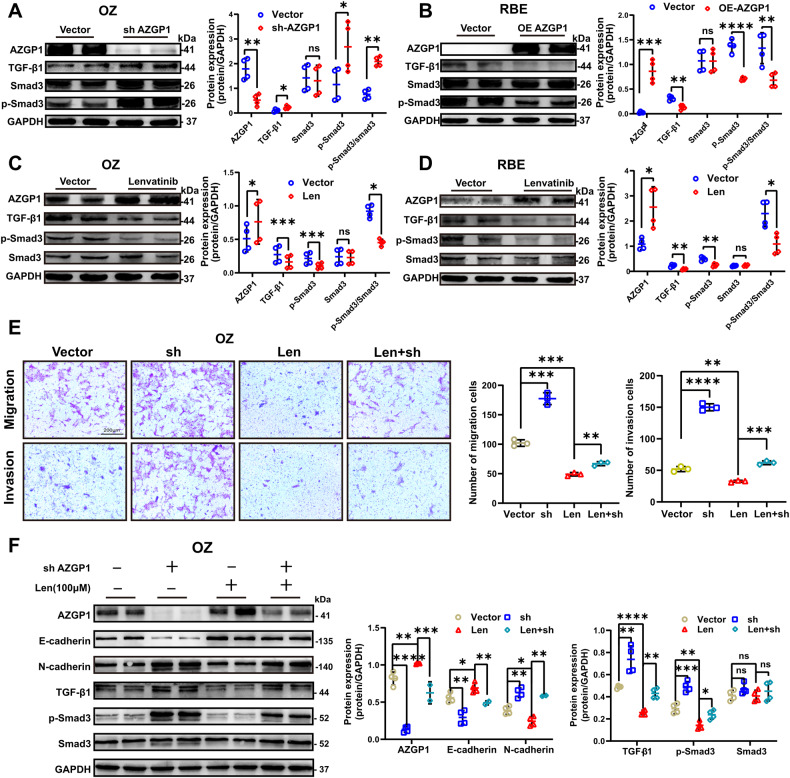
Lenvatinib inhibits ICC EMT by mediating the TGF-β1/Smad3 pathway through AZGP1. **A** TGF-β1, Smad3, and p- Smad3 protein expression levels in OZ cells after AZGP1 knockdown were detected by Western blotting. **B** TGF-β1, Smad3, and p- Smad3 protein expression levels in RBE cells after AZGP1 overexpression were detected by Western blotting. **C**, **D** The protein expression levels of AZGP1, TGF-β1, Smad3, and p-Smad3 in OZ and RBE cells treated with lenvatinib were detected by Western blotting. **E** The results of the transwell assay showed that lenvatinib reversed the migration and invasion induced by AZGP1 knockdown. **F** Lenvatinib reverses EMT and TGF-β1/Smad3 signaling pathway activation induced by AZGP1 knockdown. ns, nonsignificant. *p < 0.05, **p < 0.01, ***p < 0.001, and ****p < 0.0001.

We explored the specific role of AZGP1 in the inhibition of ICC-EMT by lenvatinib. First, we found that AZGP1 could regulate the TGF-β1/Smad3 pathway (Fig. 5A, B). Subsequently, we found that lenvatinib upregulated AZGP1 and inhibited the TGF-β1/Smad3 pathway (Fig. 5C, D). In addition, to investigate whether lenvatinib inhibited EMT in ICC cells by activating AZGP1, we found that downregulated AZGP1 expression could partially restore the migration and invasion inhibited by the addition of lenvatinib (Fig. 5E). Furthermore, we found that downregulated AZGP1 expression could partially restore the EMT and TGF-β1/Smad3 signaling pathways inhibited by the addition of lenvatinib (Fig. 5F). Based on our results, lenvatinib may directly or indirectly inhibit TGF-β1/Smad3 signaling by activating AZGP1, thus inhibiting ICC EMT.

### Lenvatinib promotes AZGP1 gene expression by increasing the level of H3K27Ac in the AZGP1 promoter region

**Fig. 6:**
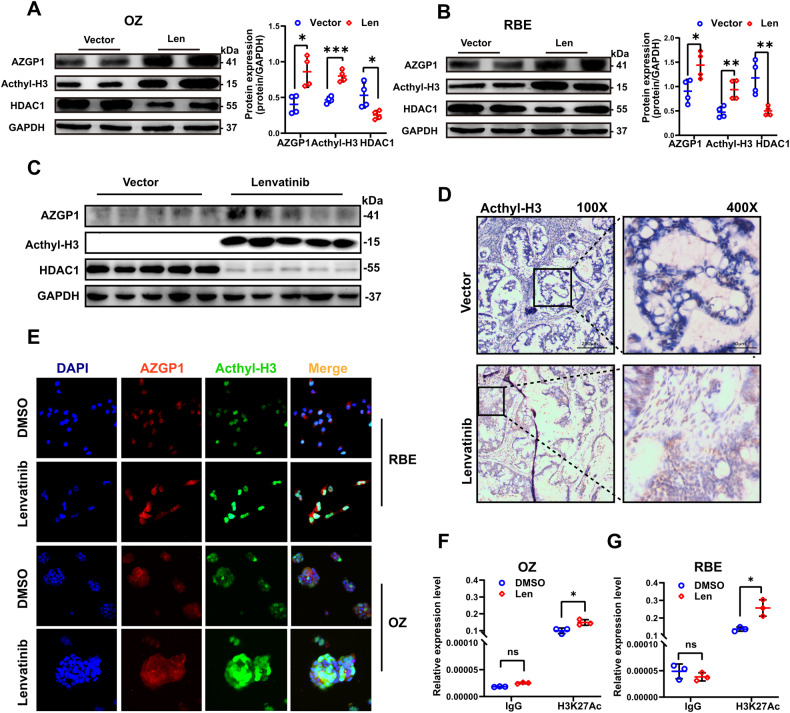
Lenvatinib modulates the expression of AZGP1 by regulating H3K27Ac. **A**, **B** The protein expression levels of H3K27Ac and HDAC1 were detected in OZ and RBE cells after lenvatinib treatment by Western blotting. **C**, **D** The protein expression levels of H3K27Ac and HDAC1 in the PDX model after lenvatinib treatment. **E** AZGP1 and H3K27Ac expression was detected in OZ and RBE cells after lenvatinib treatment using immunofluorescence. **F**, **G** The expression level of H3K27Ac in the AZGP1 promoter region of OZ and RBE cells treated with lenvatinib was detected by ChIP. ns, nonsignificant. *p < 0.05, and **p < 0.01.

Modification of histone acetylation can lead to gene activation. Lenvatinib-induced changes in AZGP1 gene expression were investigated by assessing the expression levels of acetylated histone H3 and HDACs in ICC cells. In the results, acetylated histone H3 was found to be upregulated, while HDAC1 was downregulated (Fig. 6A, B), and the levels of other HDACs were not significantly different (Supplementary Fig. S4A, S4B). The same results were obtained by Western blotting analysis (Fig. 6C) and immunohistochemical staining of ICC-PDX models (Fig. 6D). Based on these results, histone acetylation is involved the regulation of AZGP1 expression. Moreover, immunofluorescence assays showed that lenvatinib stimulated the expression of AZGP1 and acetylated histone H3 in ICC cells. Dual immunofluorescence labeling of AZGP1 and acetylated histone H3 also showed colocalization in the nucleosomes of ICC cells (Fig. 6E). Acetylation of histones frequently occurs at specific lysine residues at the N-terminus of histones, the most well-studied of which is H3K27. Therefore, we used the ChIP method to detect changes in H3K27Ac expression levels in the AZGP1 gene promoter region. We screened out DNA fragments binding to H3K27Ac and then performed qPCR using AZGP1 promoter region primers. The relative accumulation of H3K27Ac was higher in the lenvatinib group than in the control group (Fig. 6F, G, Supplementary Fig. S4C). As a result, lenvatinib enhanced H3K27Ac enrichment in the AZGP1 promoter region.

## Discussion

ICC, one of the most incurable cancers, accounts for approximately 10–15% of primary liver tumors. EMT plays a crucial role in the invasion and metastasis of ICC. Currently, there are few studies on the role of lenvatinib in ICC. Therefore, we attempted to explore the effective regulatory targets and mechanisms of lenvatinib in ICC-EMT. In this study, we found that AZGP1 was a potential key target of lenvatinib in ICC by network pharmacology and bioinformatics methods. AZGP1 is downregulated in ICC, and its reduced expression predicted a poor prognosis. The effect of lenvatinib on ICC EMT is mediated by the AZGP1/TGF-β1/Smad3 pathway. Finally, lenvatinib may activate AZGP1 by increasing H3K27Ac levels in the AZGP1 promoter region.

In this study, we first used the PharmMapper Server reverse screening method to screen potential lenvatinib targets. PharmMapper Server, based on the effect group stereostructure of drugs, is an important target computing server to screen potential specific targets; it can find the best mapping posture between drug molecules and potential targets. It has been applied to predict targets of treatments in different disease fields, such as compound Salvia miltiorrhiza prescription in the treatment of cardiovascular diseases [39], trichlorodibenzofuran in the treatment of breast cancer [40], ginsenoside Rh2 in metastatic osteosarcoma [41], saffron [42] and cardamom essential oil [43]. By means of bioinformatics methods, we found that AZGP1 is the most relevant key target of lenvatinib in ICC.

Second, we explored the expression of AZGP1 in ICC tissues and further studied its clinical significance. Researchers have identified AZGP1 as a tumor suppressor in several types of cancer, including hepatocellular carcinoma [23, 25, 26], pancreatic cancer [24] and gastric cancer [21, 27]. However, the expression of AZGP1 has not been reported in ICC. When we analyzed tissue samples from 74 ICC patients, we found that 72.97% of tumor tissues had reduced or absent AZGP1 expression, which was consistent with the results in the TCGA and GEO databases. According to survival analysis, ICC patients with low expression of AZGP1 had a shorter life expectancy, which was also found in the TCGA and GEO data analyses. AZGP1 is a prognostic factor in several malignant tumors [20, 21, 44–46]. Loss or attenuation of AZGP1 expression has been shown to be associated with reduced RFS and metastatic survival in prostate cancer patients [47, 48]. In the present study, according to Cox regression analysis, AZGP1 was an independent predictor of survival in ICC patients. These results support the potential of AZGP1 as a novel prognostic biomarker for ICC.

Next, we studied the biological function of AZGP1 in tumors. Huang Tian et al. found that AZGP1 can regulate cancer function by regulating PTEN in hepatocellular carcinoma [26]. Li Wenbo et al. found that AZGP1 reduced the proliferation and apoptosis of gastric cancer cells and the progression of gastric cancer [27]. Studies have also shown that AZGP1 inhibited EMT in hepatocellular carcinoma by blocking the TGF-β1/ERK2 pathway [25] and TGF-β1/ERK 2-induced EMT in pancreatic cancer [24]. Our study showed that reducing AZGP1 levels promoted the proliferation, migration, invasion, and EMT of ICC cells, possibly by activating the TGF-β1/Smad3 signaling pathway and promoting EMT in ICC cells.

Although lenvatinib has first-line treatment status for advanced hepatocellular carcinoma, the effect of lenvatinib on ICC has rarely been reported. In this study, we examined the effects of lenvatinib on ICC cells and found that lenvatinib inhibited ICC cell growth in a concentration-dependent manner. Migration and invasion are key factors in ICC development. Based on the results of this study, we found that lenvatinib inhibited EMT in ICC, thereby inhibiting the migration and invasion of ICC. We also tested the therapeutic effects of lenvatinib in the ICC-PDX model. Direct implantation of human primary tumor cells into immunodeficient mice was used to establish a PDX model [49, 50], which showed morphology, histology and immunoreactivity similar to the tumor of the primary patient [51], making it an excellent model for testing antitumor drugs. Based on the PDX model, we assessed lenvatinib’s effectiveness in treating ICC, and the results were consistent with previous research findings [16]. Lenvatinib seems to inhibit the proliferation, migration, invasion, and EMT of ICC. Our study then focused on the mechanism of action of lenvatinib in ICC-EMT. Lenvatinib inhibits ICC-EMT by downregulating TGF-β1 and thereby decreasing the level of phosphorylated Smad3.

It is unclear how lenvatinib influences the expression of AZGP1 to affect the prognosis of ICC patients. According to the present study, AZGP1 expression is upregulated following lenvatinib exposure in ICC cells, which was also observed in the ICC-PDX model. This phenomenon led us to speculate that the effect of lenvatinib on ICC-EMT was inhibited by increased AZGP1 expression and TGF-β1/Smad3 signaling. We then tested this hypothesis and found that lenvatinib increased AZGP1 expression and inhibited TGF-β1 expression, Smad3 phosphorylation, and EMT-dependent AZGP1 expression in ICC cells by increasing AZGP1 expression.

We also examined the molecular mechanism by which lenvatinib may increase AZGP1 expression in ICC cells. The methylation of DNA and modification of histones have profound effects on chromosome stability and gene transcription [52]. A study found that the loss of AZGP1 expression was caused by histone deacetylation in pancreatic ductal adenocarcinoma [24]. Histone deacetylation has also been reported to regulate the expression of AZGP1 in hepatocellular carcinoma [26]. Acetylation of histone H3K27 is a marker of gene activation and expression. There is evidence that esophageal cancers have higher levels of H3K27Ac in the CCAT1 promoter region, which may explain the increased expression of CCAT1 [53], and the increased H3K27 acetylation level in the GHET1 promoter region in liver cancer may be the cause of the increased GHET1 expression [54]. This study revealed that lenvatinib increases the expression of AZGP1 by activating H3K27Ac at the promoter region of the AZGP1 gene, thus inhibiting ICC cell migration, invasion, and EMT.

**Fig. 7:**
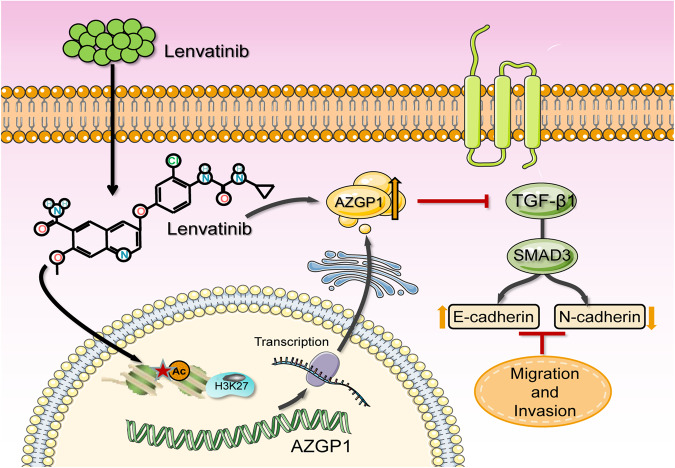
Schematic diagram of lenvatinib inhibition of EMT in ICC cells. Lenvatinib enhanced AZGP1 activity through H3K27Ac modification and inhibited the downstream TGF-β1/Smad3 signaling pathway.

In conclusion, our study revealed that AZGP1 is the key target of lenvatinib in ICC, and its low expression in ICC cancer tissues is associated with a poor prognosis in patients. The underlying mechanisms of the effects of lenvatinib on ICC are shown in Fig. 7. Lenvatinib inhibits the TGF-β1/Smad3 pathway by activating AZGP1 expression and reversing the EMT progression of ICC caused by AZGP1 deletion. Lenvatinib can restore AZGP1 function by increasing H3K27Ac levels in the AZGP1 promoter region. In summary, lenvatinib reverses EMT by upregulating AZGP1 expression, which may be a promising treatment for ICC.

## Supplementary information

Supplementary information

Original Data File

aj-checklis

## Data Availability

The data used to support the findings of this study are available from the corresponding author on request.
