# Supplementary material for: AZGP1 activation by lenvatinib suppresses intrahepatic cholangiocarcinoma epithelial-mesenchymal transition through the TGF-β1/Smad3 pathway
Source: Cell Death Dis. 2023 Sep 5;14(9):590. doi: 10.1038/s41419-023-06092-5 (PMC10480466; doi:10.1038/s41419-023-06092-5)
Supplement: Supplementary file 1 — Supplementary information [file 41419_2023_6092_MOESM1_ESM.docx]

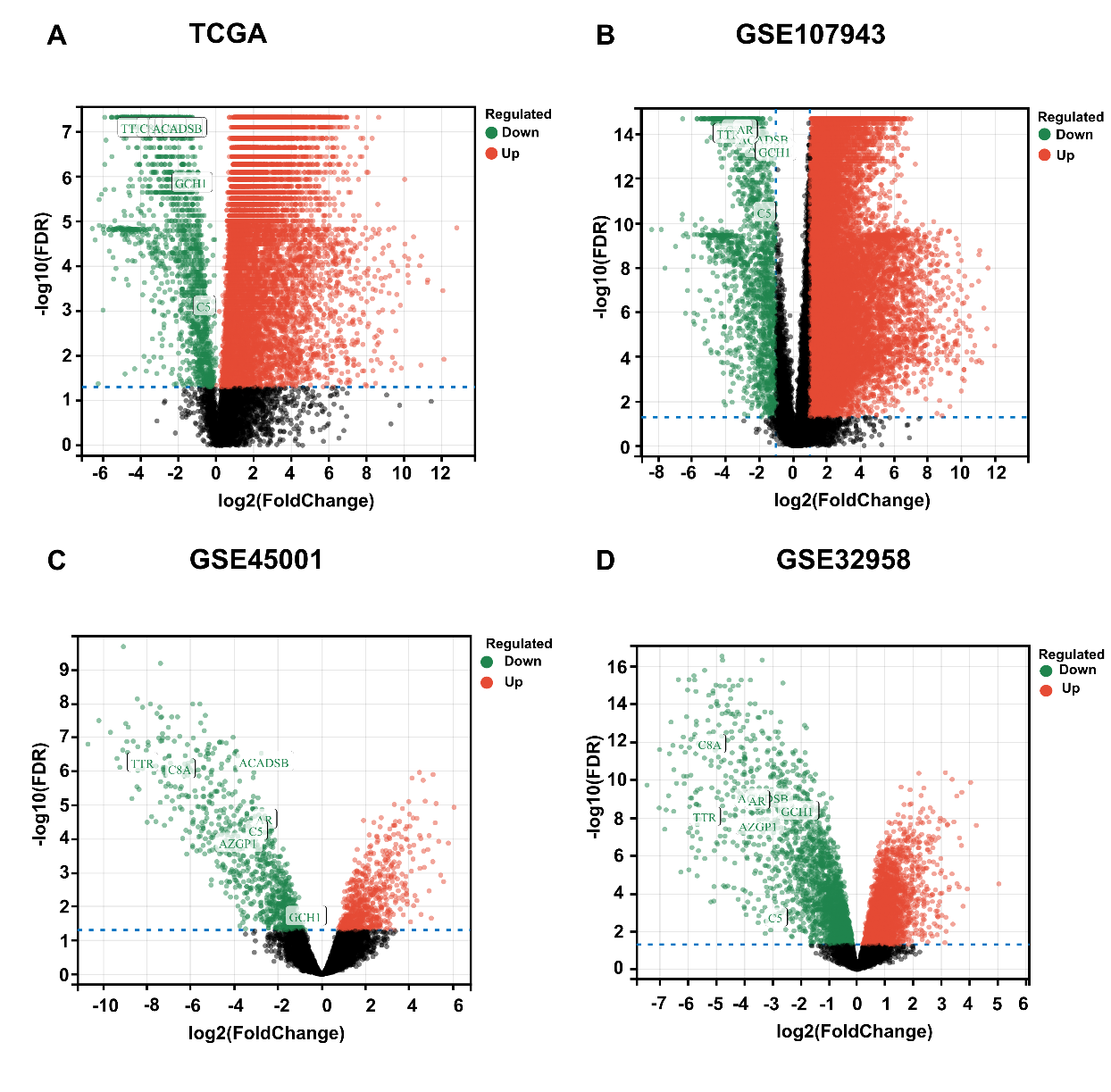


**Supplementary Fig. 1.** **Differential gene volcanic map in TCGA, GSE107943, GSE45001, and GSE32958 datasets.** (A) Volcano plot of 12144 DEGs in TCGA. (B) Volcano plot of 12077 DEGs in GSE107943. (C) Volcano plot of 1510 DEGs in GSE45001. (D) Volcano plot of 2272 DEGs in GSE32958.


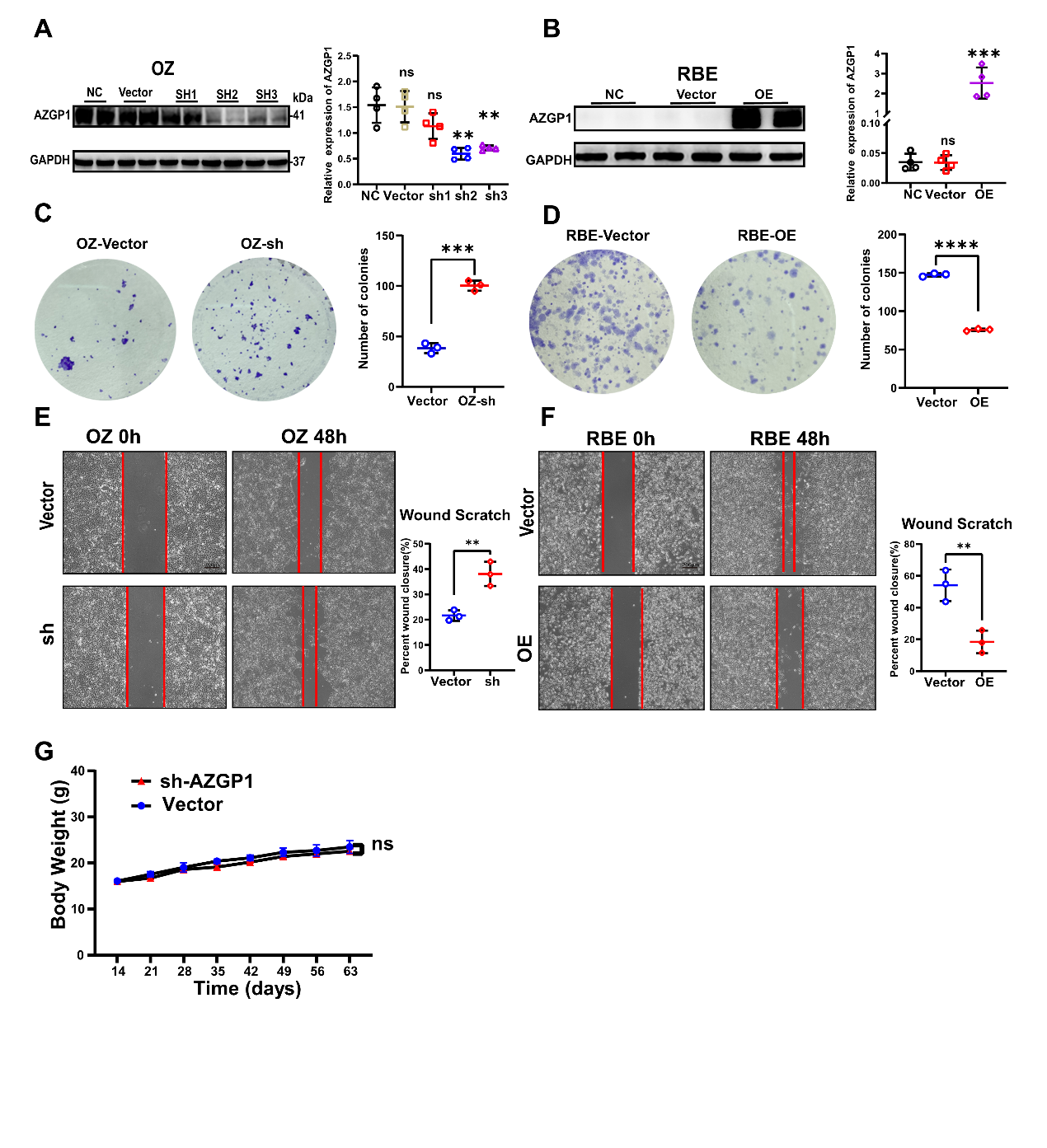


**Supplementary Fig. 2. AZGP1 regulates ICC cell proliferation and migration.** (A) OZ cell lines with stable knockdown of AZGP1 were constructed. (B) RBE cell lines with stable overexpression of AZGP1 were constructed. (C) The results of the colony formation assay demonstrated that knockdown of endogenous AZGP1 in OZ cells promoted cell proliferation. (D) The results of the colony formation assay showed that the overexpression of endogenous AZGP1 in RBE cells inhibited cell proliferation. (E) The results of the wound scratch assay demonstrated that knockdown of endogenous AZGP1 in OZ cells promoted cell migration. (F) The results of the wound scratch assay showed that the overexpression of AZGP1 in RBE cells inhibited cell migration. (G) Weight of PDX model mice. ns, nonsignificant. *p < 0.05, **p < 0.01, ***p < 0.001, and ****p < 0.0001.

**
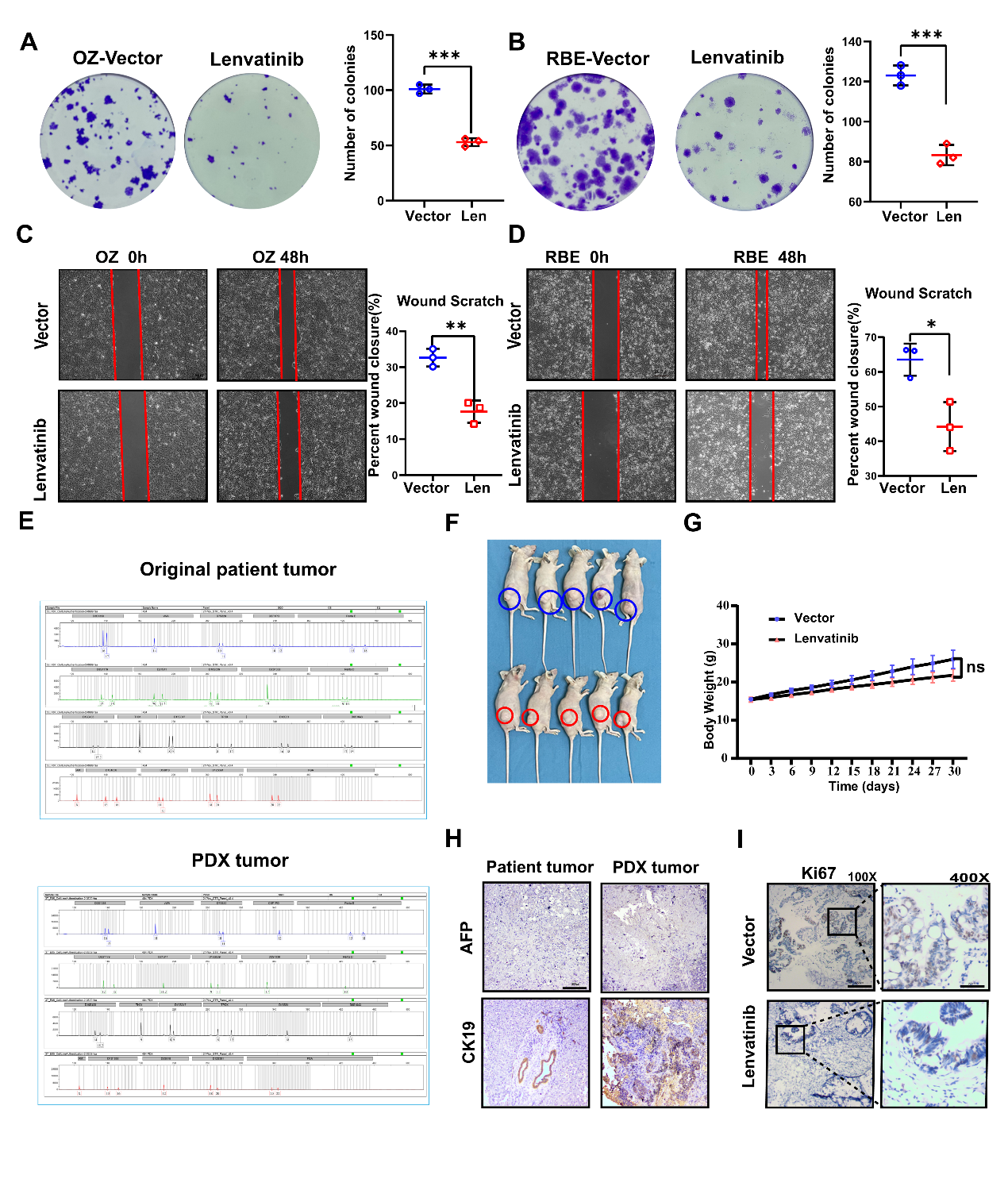
**

**Supplementary Fig. 3. Lenvatinib inhibits ICC proliferation and migration.** (A, B) Lenvatinib inhibited the proliferation of OZ and RBE cells in colony formation assays. (C, D) Lenvatinib inhibited the migration of OZ and RBE cells in the wound scratch assay. (E) Short tandem repeat profiling. (F) The PDX tumors in the lenvatinib group were smaller than those in the control group. (G) Weight of PDX model mice. (H) Immunohistochemical analysis of AFP and CK19 expression in tumor tissue of the patient and PDX model. (I) Immunohistochemical analysis of Ki-67 expression in the PDX model. ns, nonsignificant, *p < 0.05, **p < 0.01, ***p < 0.001, and ****p < 0.0001.


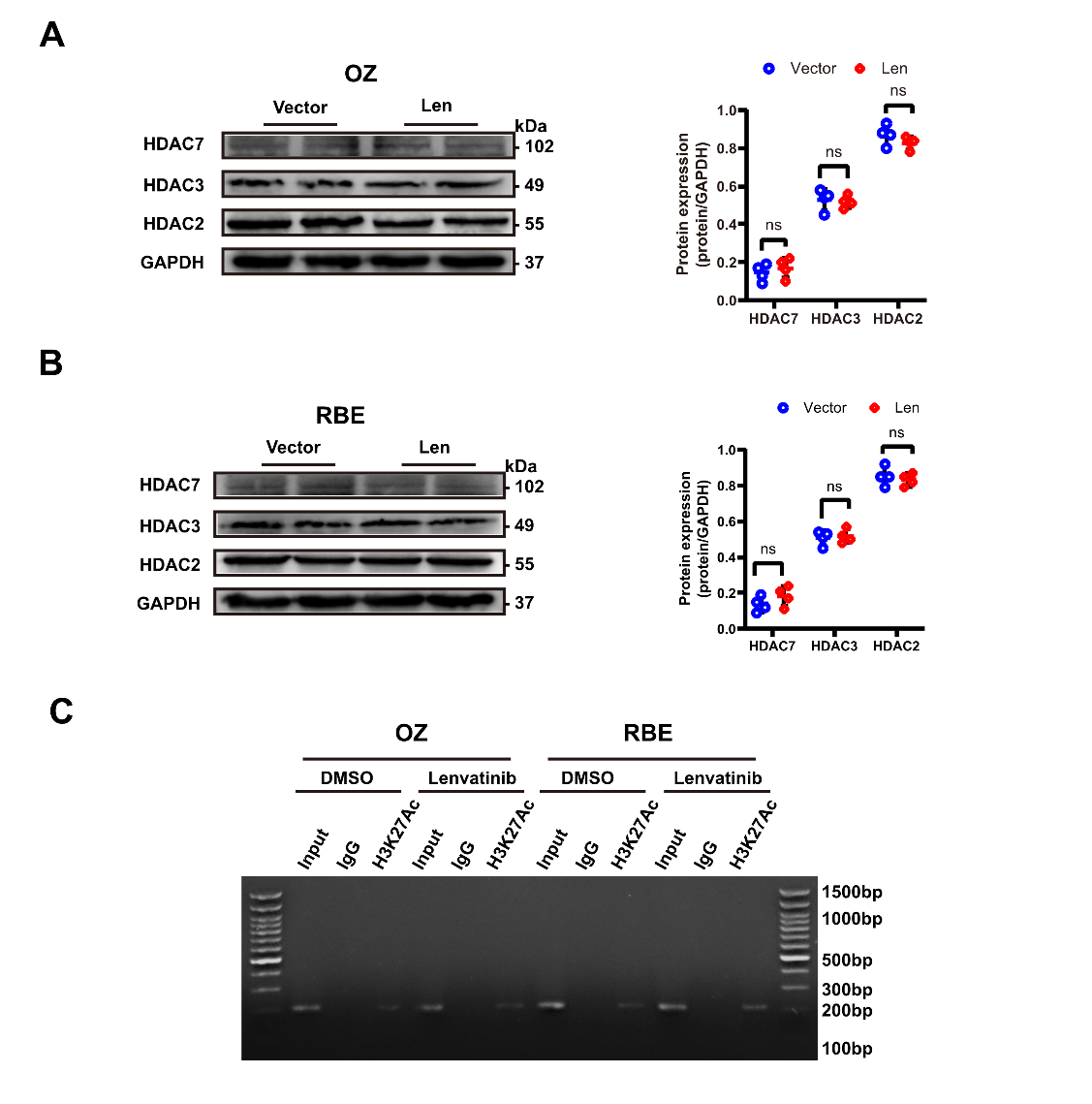


**Supplementary Fig. 4.** **Lenvatinib regulates AZGP1 expression through acetylation.** (A, B) The protein expression levels of HDAC2, HDAC3 and HDAC7 were detected in OZ and RBE cells after lenvatinib treatment by Western blotting. (C) Representative images of CHIP assays.
