# Supplementary material for: AZGP1 activation by lenvatinib suppresses intrahepatic cholangiocarcinoma epithelial-mesenchymal transition through the TGF-β1/Smad3 pathway
Source: Cell Death Dis. 2023 Sep 5;14(9):590. doi: 10.1038/s41419-023-06092-5 (PMC10480466; doi:10.1038/s41419-023-06092-5)
Supplement: Supplementary file 2 — Original Data File [file 41419_2023_6092_MOESM2_ESM.docx]

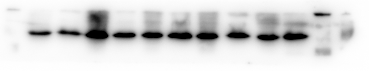


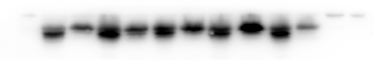


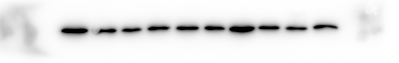


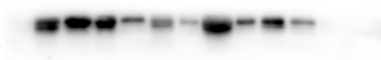


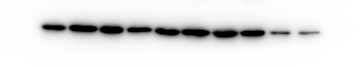


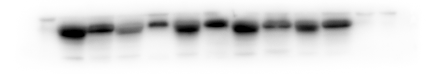


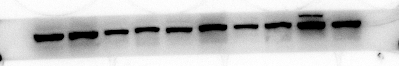


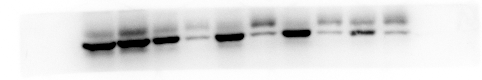


Figure S5. original western blots of Figure 2B.


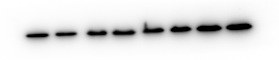


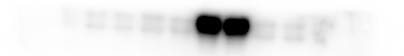


Figure S6. original western blots of Figure 3B.


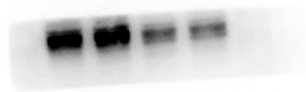


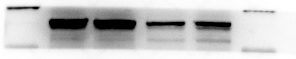


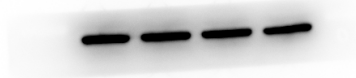


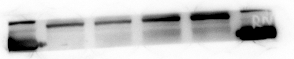


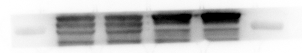


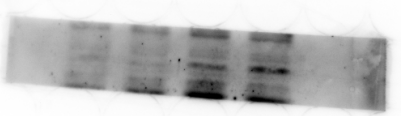


Figure S7. original western blots of Figure 3E.


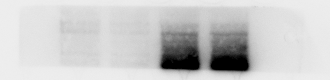


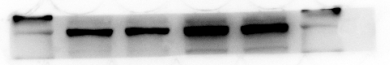


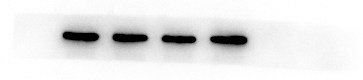


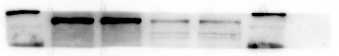


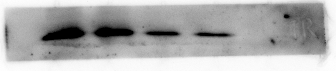


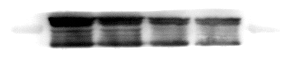


Figure S8. original western blots of Figure 3F.


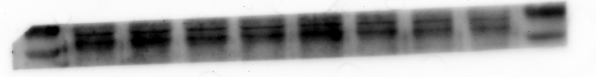
.


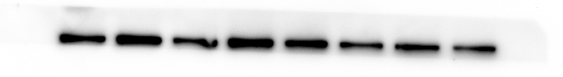


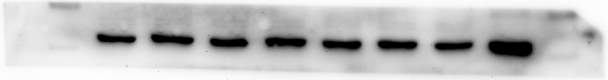


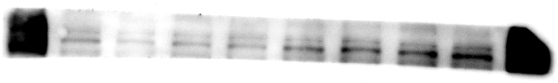


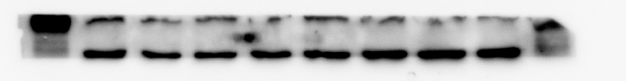


Figure S9. original western blots of Figure 3J.


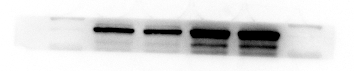


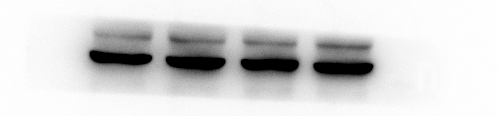


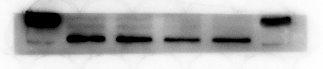


Figure S10. original western blots of Figure 4E.


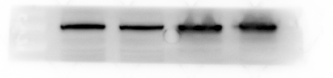


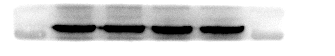


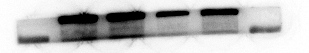


Figure S11. original western blots of Figure 4F.


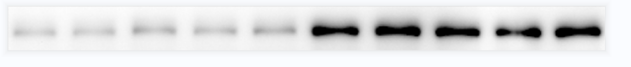


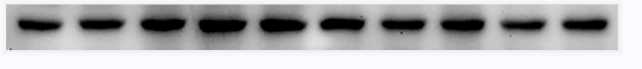


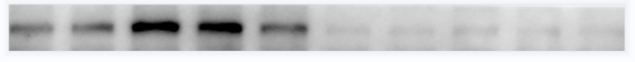


Figure S12. original western blots of Figure 4N.


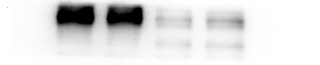


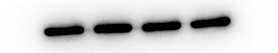


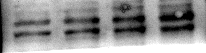


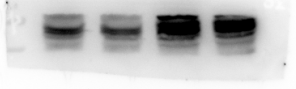


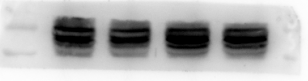


Figure S13. original western blots of Figure 5A.


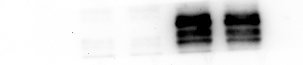


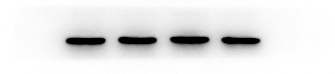


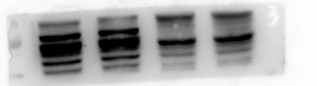


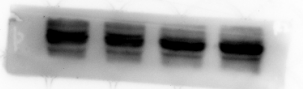


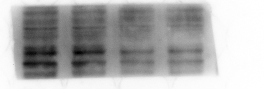


Figure S14. original western blots of Figure 5B.


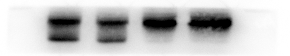


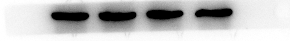


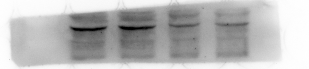


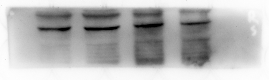


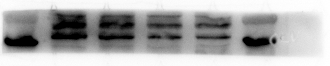


Figure S15. original western blots of Figure 5C.


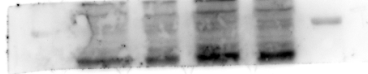


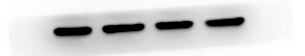


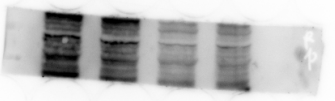


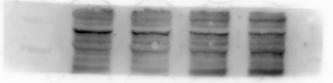


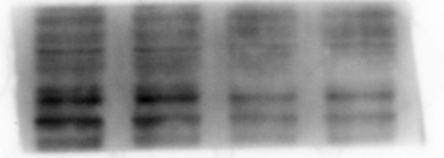


Figure S16. original western blots of Figure 5D


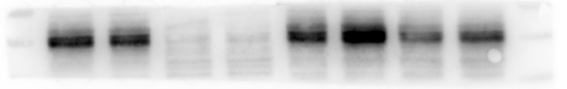
.


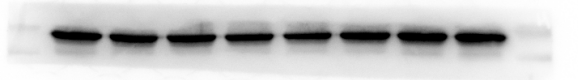


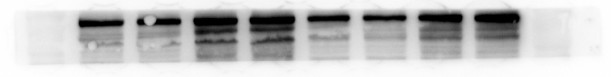


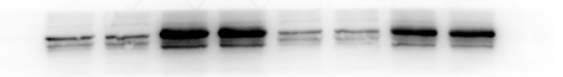


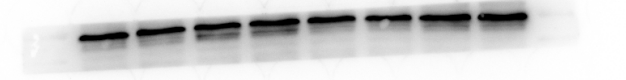


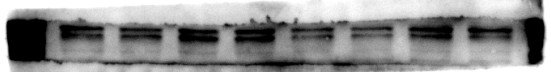


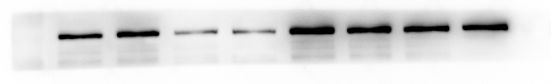


Figure S17. original western blots of Figure 5F


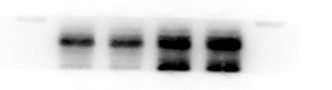


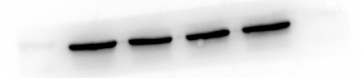


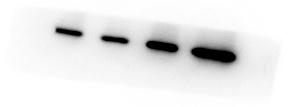


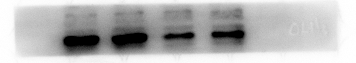


Figure S18. original western blots of Figure 6A


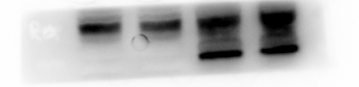


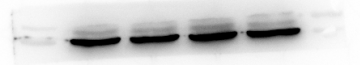


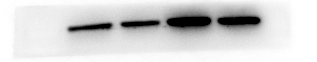


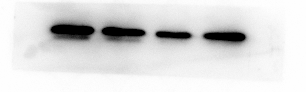


Figure S19. original western blots of Figure 6B


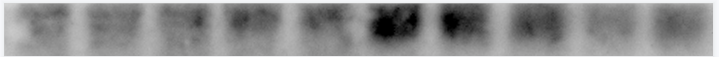


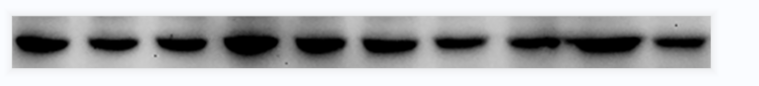


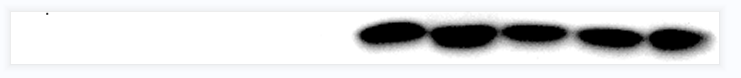


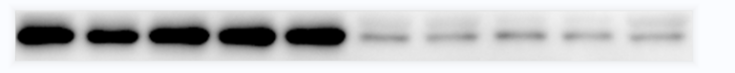


Figure S20. original western blots of Figure 6C


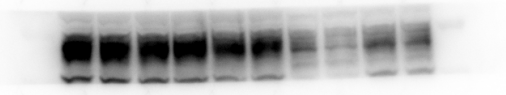


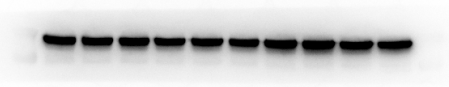


Figure S21. original western blots of Supplementary-Figure 2A


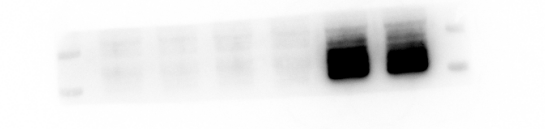


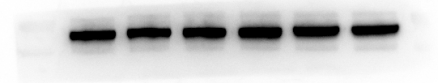


Figure S22. original western blots of Supplementary-Figure 2B


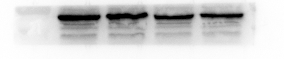


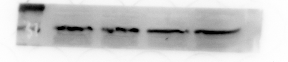


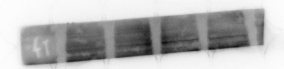


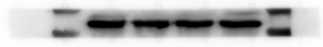


Figure S23. original western blots of Supplementary-Figure 4A


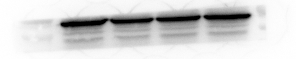


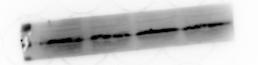


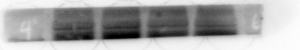


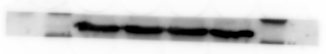


Figure S24. original western blots of Supplementary-Figure 4B
